# Analysis of the short- and long-term outcomes of membrane anatomy-guided laparoscopic spleen-preserving circumferential splenic hilar lymph node dissection for treating advanced proximal gastric cancer

**DOI:** 10.1186/s12957-019-1610-z

**Published:** 2019-04-09

**Authors:** Yanchang Xu, Zhixiong Li, Guofeng Pan, Haiyan Wu, Wenlin Lin, Jian Chen, Junpeng Li

**Affiliations:** 1 Gastrointestinal Surgery Unit 1, Teaching Hospital of Putian First Hospital of Fujian Medical University, Putian, 351100 Fujian China; 2 Department of Pathology, Teaching Hospital of Putian First Hospital of Fujian Medical University, Putian, 351100 Fujian China

**Keywords:** Mesenteric anatomy, Laparoscopy, Advanced proximal gastric adenocarcinoma, D2 lymph node dissection, Splenic hilar lymph node circumferential dissection

## Abstract

**Objective:**

To investigate the short- and long-term efficacy of membrane anatomy-guided laparoscopic spleen-preserving circumferential splenic hilar lymph node dissection for the treatment of advanced proximal gastric cancer.

**Methods:**

A retrospective analysis was conducted in 186 patients with advanced proximal gastric cancer who underwent mesenteric anatomy-guided laparoscopic spleen-preserving splenic hilar lymph node dissection for advanced proximal gastric cancer in our center from March 2013 to March 2018. The patients were divided into two groups: one group was the laparoscopic anterior splenic hilar lymph node dissection group which we named *L-ASHD*, *n* = 103), while the other group was the laparoscopic circumferential splenic hilar lymph node dissection group which we named *L-CSHD*, *n* = 83).

**Results:**

There was no significant difference in total operative time, intraoperative blood loss, postoperative length of hospital stay, and incidence of postoperative complications, etc. (*P* > 0.05). The number of harvested splenic hilar lymph nodes and the number of patients with harvested positive splenic hilar lymph nodes were both higher in the L-CSHD than in the L-ASHD (3.90 ± 2.52 vs. 3.02 ± 3.07, *P* < 0.05; 19 vs. 9 patients, *P* < 0.05). The positive rate of lymph nodes behind the splenic hilar was 8.4%. Kaplan–Meier survival curves showed that patients in the L-CSHD had similar OS and DFS compared with those of patients in the L-ASHD.

**Conclusion:**

Membrane anatomy-guided laparoscopic spleen-preserving circumferential splenic hilar lymph node dissection for advanced proximal gastric cancer is safe and feasible and can help avoid the incomplete dissection of positive lymph nodes.

**Electronic supplementary material:**

The online version of this article (10.1186/s12957-019-1610-z) contains supplementary material, which is available to authorized users.

Gastric cancer is the most common malignant tumor of the digestive tract worldwide. Its morbidity and mortality rate are among the highest. According to the latest global cancer data published in the CA Journal, there were nearly one million new cases of gastric cancer and more than 700,000 deaths due to gastric cancer worldwide in 2012 [1]. In China, the rate of early detection of gastric cancer is low due to the low rate of gastroscopy screening. Approximately 80–90% of patients with a confirmed diagnosis of gastric cancer were in an advanced gastric cancer stage and thus had poor prognosis. Advanced proximal gastric cancer is commonly associated with No. 10 lymph node metastasis. The lymph node metastasis rate reported in the literature is 9.8–20.9% [2]. The 14th edition of the Japanese *Gastric Cancer Treatment Protocol* clearly specifies that splenic hilar lymph node dissection is required for the treatment of advanced proximal gastric cancer [3]. To date, the procedure of laparoscopic spleen-preserving No. 10 lymph node dissection is available at only a few centers in China. Moreover, only anterior splenic hilar lymph node dissection is available at these centers. Therefore, in many cases, splenic hilar lymph nodes may not be completely removed. As a result, positive lymph nodes may be missed. To ensure the thoroughness of splenic hilar lymph node dissection, we recently proposed for the first time the surgical concept of mesenteric anatomy-guided laparoscopic spleen-preserving circumferential splenic hilar lymph node dissection and have applied it in our clinical practice. In this work, we summarize the short-term efficacy of this surgical procedure and assess its safety and feasibility.

## Material and Methods

### General data

A retrospective analysis of 186 patients with advanced proximal gastric cancer who underwent mesenteric anatomy-guided laparoscopic spleen-preserving splenic hilar lymph node dissection for advanced proximal gastric cancer in the Gastrointestinal Surgery Unit 1 of the Putian First Hospital in Fujian Province from March 2013 to March 2018 was conducted. One group of patients (the *L-ASHD*, *n* = 103) underwent only anterior splenic hilar lymph node dissection, while another group of patients (the *L-CSHD*, *n* = 83) underwent circumferential splenic hilar lymph node dissection, i.e., routine anterior splenic hilar lymph node dissection plus posterior splenic hilar lymph node dissection. All procedures were performed by an experienced surgeon who had performed more than 500 laparoscopic radical gastrectomies. The surgical procedure and the standards of lymph node dissection were based on the 3rd edition of the *Japanese Gastric Cancer Treatment Guidelines* 2010 (Physicians’ Edition, May 2010). Total gastrectomy and D2 lymph node dissection (including spleen-preserving No. 10 lymph node dissection) were performed. TNM staging followed the staging system described in the 7^th^ edition of the *American Joint Committee on Cancer* (AJCC).

### Patient selection criteria

#### Inclusion criteria:

1. Patients between 18 and 78 years of age.
2. Histopathological diagnosis of gastric primary lesions was gastric adenocarcinoma (papillary adenocarcinoma, tubular adenocarcinoma, mucinous adenocarcinoma, signet-ring cell carcinoma and poorly differentiated adenocarcinoma).
3. Preoperative clinical staging indicated locally advanced proximal stomach cancer (cT2-4a, N-/+, M0).
4. No distant metastasis or direct invasion of the surrounding organs was evident prior to surgery.
5. Preoperative Eastern Cooperative Oncology Group (ECOG) performance score was 0/1.
6. Preoperative American Society of Anesthesiologists (ASA) score was between I and III.

#### Exclusion criteria:

1. Pregnant or lactating women.
2. Patients suffering from severe mental illness.
3. Patients with history of upper abdominal surgery (with exception of laparoscopic cholecystectomy).
4. Preoperative imaging studies showed locally matted/fused or enlarged lymph nodes (maximum diameter ≥ 3 cm) including obviously enlarged or matted/fused No. 10 lymph nodes.
5. Patients undergoing neoadjuvant treatment.
6. Patients undergoing sustained systemic corticosteroid treatment within 1 month prior to surgery.
7. Patients who had gastric cancer complications (bleeding, perforation, or obstruction) and required emergency surgery.
8. Imaging indicates the patients who required splenectomy due to obvious tumor invasion of the spleen and the splenic vessels

#### Rejection criteria:

1. Patients with confirmed M1 during or after surgery: No distant metastasis was evident on preoperative examination, but intraoperative exploration/postoperative pathology confirmed the presence of distant metastases; postoperative peritoneal lavage cytological test result was positive.
2. Intraoperative/postoperative study confirmed tumors that were at stage T4b or invading the duodenum.
3. Intraoperative findings showed uncertainty of R0 resection due to regionally matted/fused lymph nodes or unresectable lymph nodes due to the encirclement of vital vessels.
4. Intraoperative discovery that the patients who required splenectomy due to obvious tumor invasion of the spleen and the splenic vessels.

### Follow-up

Specially trained researchers were called to follow-up with patients after their operation by using outpatient records, telephone calls, letters, and visitation every 3 months for 2 years and then every 6 months from postoperative years 3 to 5. Survival time was defined as the time from surgery to either death or the final follow-up date of February 2019.

### Surgical procedure

The four-step procedure of laparoscopic spleen-preserving circumferential splenic hilar lymph node dissection for proximal gastric cancer is described below (Additional file 1: Movie S1).


**Additional file 1: Movie S1.** Membrane anatomy-guided Laparoscopic spleen-preserving circumferential splenic hilar lymph node dissection for treating advanced proximal gastric cancer. (MP4 168246 kb)


**Fig. 1:**
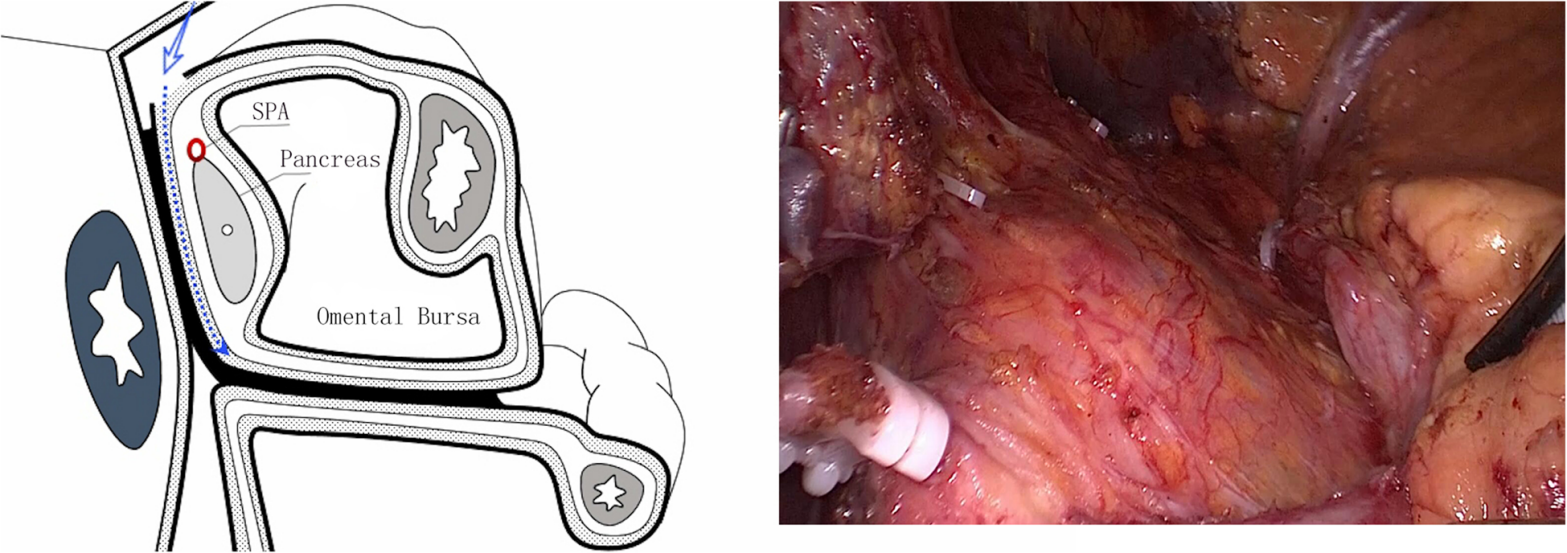
Exposure of the posterior-superior space of the pancreas

Step 1: Exposure of the posterior-superior space of the pancreas. The anterior pancreatic fascia was incised to access the origin of the splenic artery along the upper edge of the pancreatic parenchyma. The separation was continued posteriorly along the surface of the splenic artery until it reached the upper edge of the splenic vein or the posterior part of the pancreas (i.e., entering the posterior-superior space of the pancreas); the separation was then continued in the posterior-superior space of the pancreas to expose the splenic arterial segment in the pancreas, i.e., to expose the anterior space of Toldt’s fascia, which forms from the posterior pancreatic fascia and the posterior peritoneum (Fig. 1). This space is a loose, avascular space. Via this space, lymph node dissection can be performed to completely remove the No. 11p and No. 11d lymph nodes, with separation to the left, posterior, and superior sides until the pancreatic body, the upper pole of the spleen, and the anterior fascia of the left diaphragm, respectively, are reached.

**Fig. 2:**
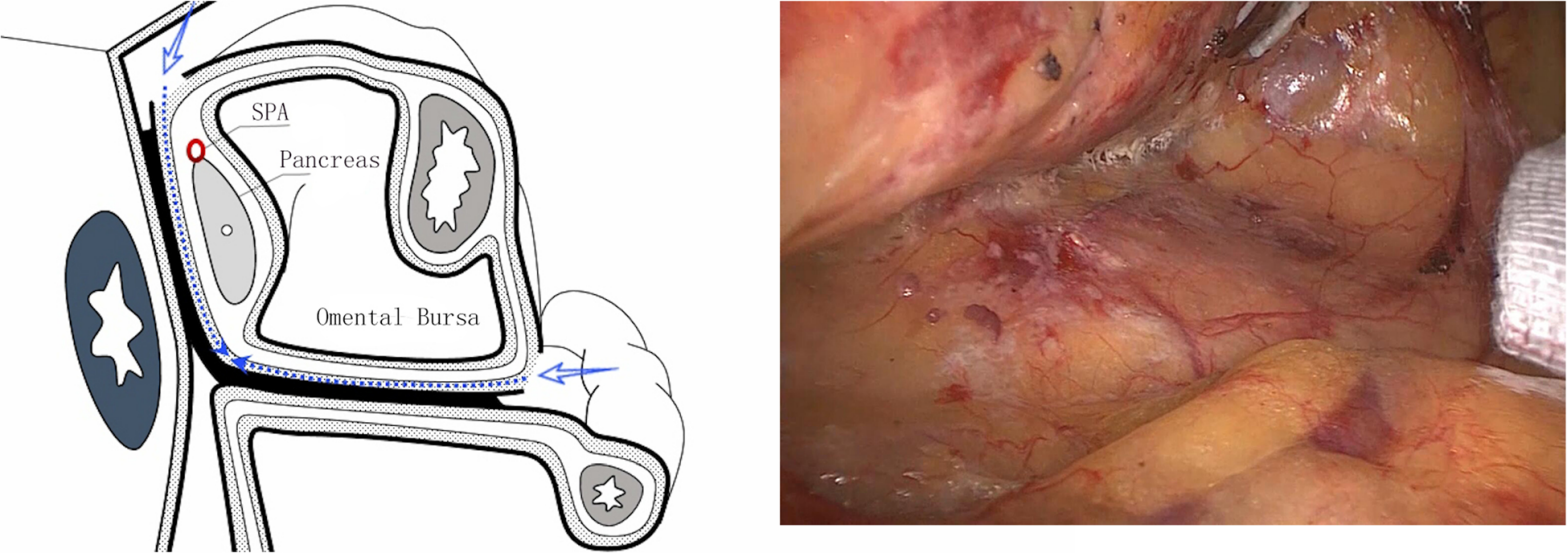
Expose the posterior-inferior pancreatic space

Step 2: Expose the posterior-inferior pancreatic space. The attached edge of the mesogastrium and the transverse mesocolon was incised to access the space between them. To enter the posterior-inferior pancreatic space, the separation was continued towards the posterior-inferior part of the pancreas to the lower pole of the spleen laterally and the body of the pancreas medially; the separation was then continued superiorly to meet the posterior-superior space of the pancreas described in step 1 (Fig. 2).

**Fig. 3:**
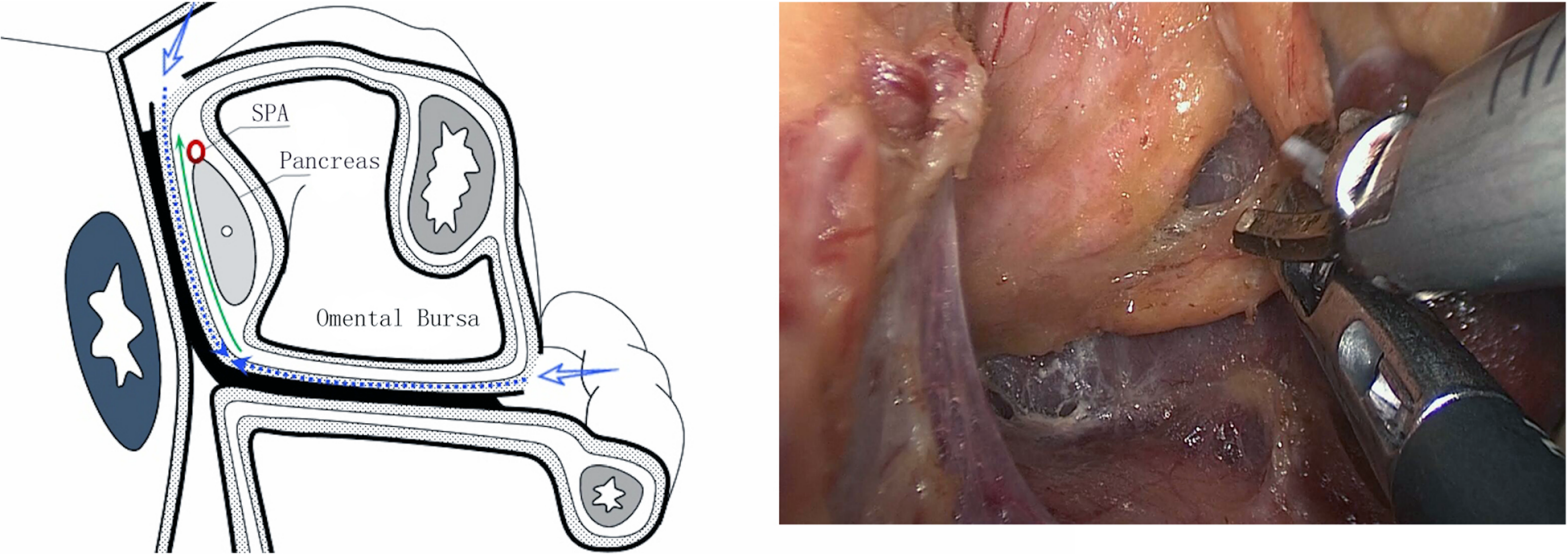
Exposure of the posterior splenic hilum

Step 3: Exposure of the posterior splenic hilum. Lateral area: The attachment of the mesogastrium to the inferior pole of the spleen was incised, and the mesogastrium was separated medially. Medial area: Along the posterior pancreatic tail, the attachment of the mesogastrium to the pancreas was incised, and the mesogastrium was separated laterally to meet the previously freed space. Anatomical separation of the adipose connective tissue attached to the pancreatic tail and the posterior spleen was then performed to complete the lymph node dissection in the posterior splenic hilum (Fig. 3).

**Fig. 4:**
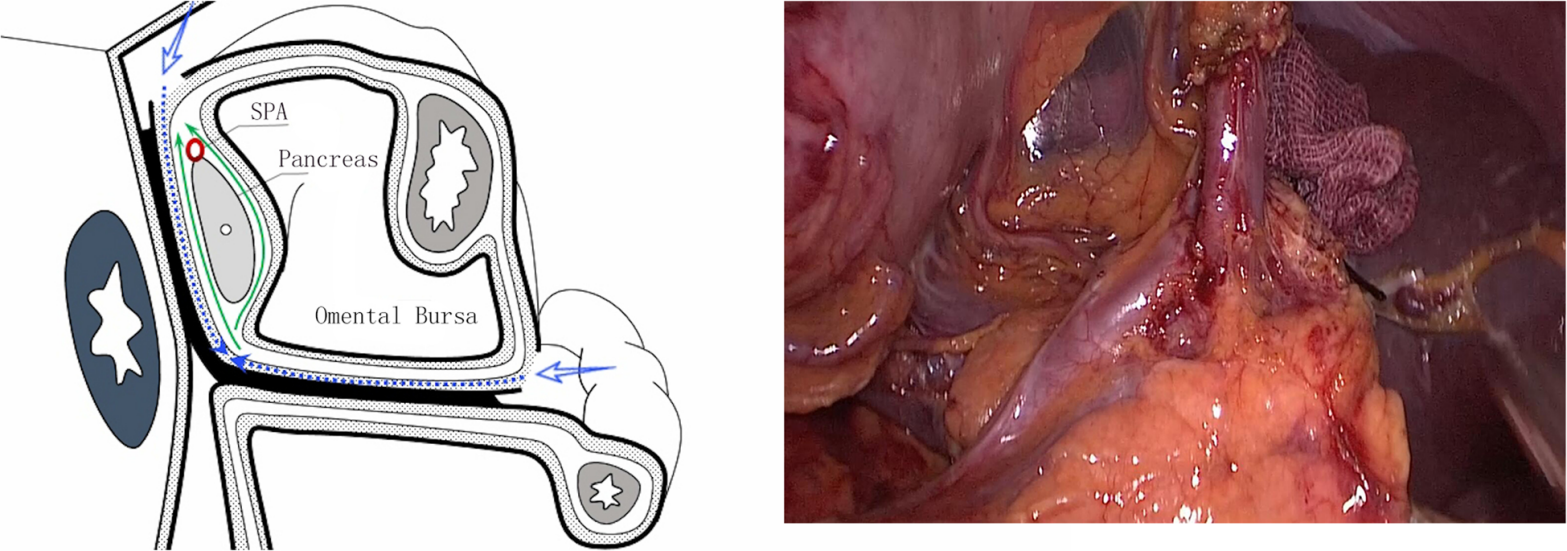
Exposure of the anterior splenic hilum


Step 4: Exposure of the anterior splenic hilum (Additional file 2: Movie S2). The anterior pancreatic fascia was incised to access the anterior space of the splenic lobar vessels. Skeletonization of each splenic lobar vessel from the proximal side to the distal side indicated completion of the lymph node dissection in the anterior splenic hilum (Fig. 4).

**Additional file 2: Movie S2.** Membrane anatomy-guided Laparoscopic spleen-preserving anterior splenic hilar lymph node dissection for treating advanced proximal gastric cancer. (MP4 370588 kb)


### Statistical analysis

SPSS 19.0 statistical software was used for statistical analysis. A *t* test was used to compare quantitative data; the mean and standard deviation are expressed as *x* ± *s*. Qualitative data were analyzed using Pearson’s *χ*^2^ test (*N* ≥ 40 and *T* ≥ 5), the continuity correction *χ*^2^ test (*N* ≥ 40 and 1 ≤ *T* < 5) or Fisher’s exact test (*n* < 40 or < 1). Cumulative survival rates were estimated by using the Kaplan–Meier method and compared with the log-rank test. *P* < 0.05 was considered to indicate statistical significance; all tests were two-tailed.

## Results

### The effect picture of laparoscopic spleen-preserving circumferential splenic hilar lymph node dissection was satisfactory

**Fig. 5:**
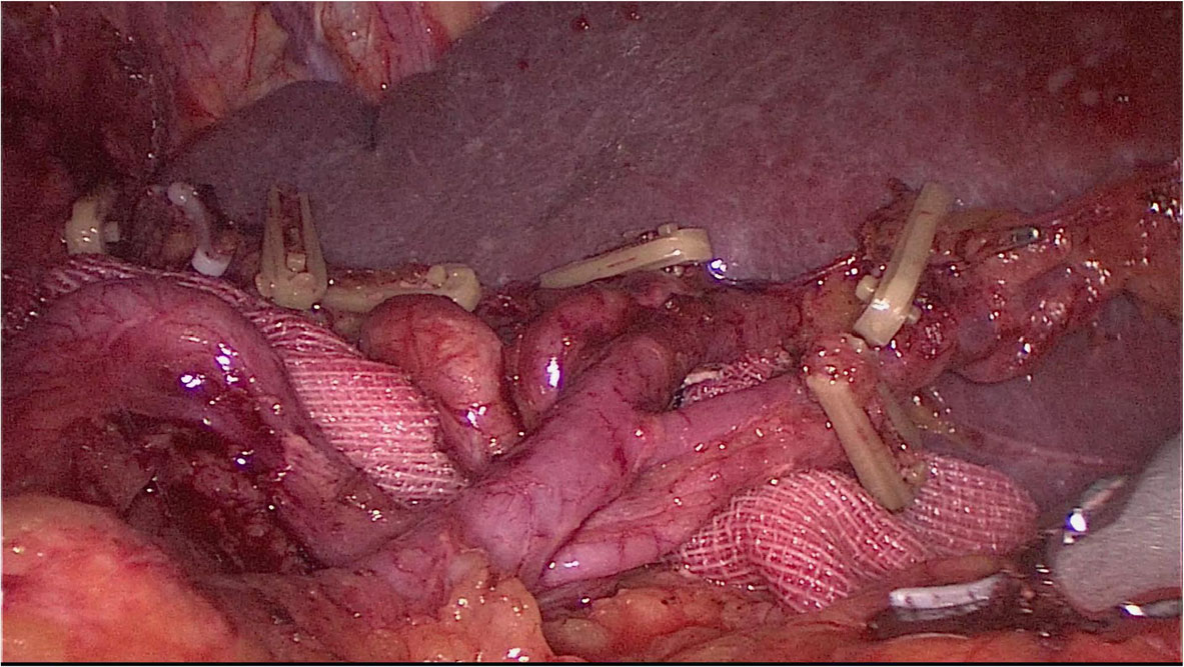
The effect picture of laparoscopic spleen-preserving circumferential splenic hilar lymph node dissection

We can see splenic hilar lymph node can be dissection completely (Fig. 5).

### Comparison of clinical baselines in the L-ASHD and the L-CSHD (Table 1)

The results show that the baseline data, including age, sex, tumor size, differentiation, and tumor stage, are comparable between the two groups of patients.

**Table 1:** Comparison of clinical baseline data in the two groups

Characteristics	L-ASHD *n* = 103	L-CSHD M − *n* = 83	*χ*^2^ value	*P* value
Age (years)	62.61 ± 9.94	63.63 ± 8.92		0.47
Sex			0.139	0.71
Male	77	64		
Female	26	19		
BMI (kg/m2)	21.81 ± 2.54	21.91 ± 2.84		0.804
Tumor diameter (cm)	4.63 ± 2.26	5.23 ± 2.64		0.101
Tumor differentiation (Lauren classification)			0.953	0.621
Intestinal type	35	23		
Mixed type	36	30		
Diffuse type	32	30		
pTNM stage			5.84	0.322
Ib	13	5		
IIa	5	8		
IIb	23	19		
IIIa	13	17		
IIIb	20	13		
IIIc	29	21		
T stage			0.763	0.683
T2	15	11		
T3	31	30		
T4	57	42		
N stage			6.883	0.076
N1	42	23		
N2	12	11		
N3	17	26		
N4	32	23		
Comorbidities			5.68	0.339
Hypertension	14	16		
Diabetes	6	3		
Coronary heart disease	3	6		
Chronic obstructive pulmonary disease (COPD)	7	3		
Silicosis	2	0		
ASA score		50	1.62	0.444
I	60	21		
II	33	12		
III	10			

*P* < 0.05 was considered statistically significant

### Comparison of perioperative data for the L-ASHD and the L-CSHD (Table 2)

The results show that there were no significant differences in total operative time, intraoperative blood loss, postoperative anal exhaust time, postoperative time of starting fluid foods, postoperative length of hospital stay, or number of harvested positive lymph nodes in the two groups (*P* > 0.05). The time required for lymph node dissection in the L-CSHD was longer than that in the L-ASHD (47.69 ± 13.02 min vs. 40.50 ± 9.46 min, *P* < 0.001), and the number of patients with harvested positive lymph nodes was higher in the former group than in the latter group (19 vs. 9 patients, *P* < 0.05). In the L-CSHD, there were 7 cases of positive lymph nodes behind splenic hilar, and the positive rate of lymph nodes behind the splenic hilar was 8.4%. The same was true for the number of harvested perigastric lymph nodes (including splenic hilar lymph nodes) (total number of harvested lymph nodes 47.83 ± 16.64 pieces vs. 37.94 ± 15.16 pieces, *P* < 0.001); the number of harvested splenic hilar lymph nodes was 3.90 ± 2.52 vs. 3.02 ± 3.07 (*P* < 0.05).

**Table 2:** Comparison of short-term clinical efficacy in the two groups

	L-ASHD *n* = 103	L-CSHD *n* = 83	*P* value
Intraoperative blood loss (ml)	97.57 ± 70.02	86.93 ± 79.20	0.332
Number of cut spleen (case)	0	0	/
Operative time (min)	263.58 ± 41.47	271.60 ± 50.95	0.238
Time for splenic hilar lymph node dissection (min)	40.50 ± 9.46	47.69 ± 13.02*	0
Total number of harvested lymph nodes (pieces)	37.94 ± 15.16	47.83 ± 16.64*	0
Number of harvested positive lymph nodes (pieces)	5.04 ± 6.69	6.53 ± 8.43	0.18
Number of patients with positive splenic hilar lymph nodes	9 (8.7%)	19 (22.3%)*	0.007
Number of harvested splenic hilar lymph nodes (pieces)	3.02 ± 3.07	3.90 ± 2.52*	0.036
Number of positive splenic hilar lymph nodes (pieces)	0.25 ± 0.92	0.46 ± 1.18	0.186
Time of first anal exhaust (days)	3.02 ± 0.71	2.90 ± 0.76	0.286
Time of starting fluid diet (days)	7.40 ± 0.82	7.12 ± 1.30	0.078
Length of hospital stay after surgery (days)	13.38 ± 2.20	12.95 ± 3.03	0.268

*P < 0.05 was considered statistically significant

### Morbidity and mortality in the L-ASHD and L-CSHD

In L-ASHD, there were eight patients that experienced intraoperative complications, giving an intraoperative morbidity rate of 7.77%. One patient experienced each of the following complications: splenic envelope injury, transverse colon injury, bleeding from the splenic artery, and bleeding from the gastric short arteries. Two patients experienced the bleeding from the gastric coronary vein, and another two patients experienced splenic infarction.

In L-CSHD, there were six patients that experienced intraoperative complications, giving an intraoperative morbidity rate of 7.23%. One patient experienced each of the following complications: splenic envelope injury, transverse colon injury, bleeding from the gastric short arteries, and bleeding from the gastric coronary vein. Two patients experienced the splenic infarction.

The results showed that there was no significant difference in the incidence of intraoperative complications in the two groups. Whatever in L-ASHD or in L-CSHD, all of the complications were treated successfully during laparoscopic surgery. No patient needed conversion to laparotomy or required splenectomy because of intraoperative injury to spleen or splenic blood vessels. For example, we treated vascular injury by laparoscopic prolene suture. We treated vascular injury by laparoscopic prolene suture and using electrocoagulation hooks to stop the bleeding to treat splenic envelope injury.

The results also showed that there was no significant difference in the incidence of postoperative complications in the L-CSHD and the L-ASHD. No perioperative deaths were reported in either group. The use of laparoscopic circumferential splenic hilar lymph node dissection did not increase surgical complications (Table 3).

**Table 3:** Comparison of intraoperative and postoperative complications in the two groups

	L-ASHD *n* = 103	L-CSHD *n* = 83	*χ*^2^ value	*P* value
Intraoperative complications (%)	8 (7.77%)	6 (7.23%)	0.019	0.89
Spleen injury	1	1		
Transverse colon injury	1	1		
Left gastric vein bleeding	2	1		
Gastric short arteries bleeding	1	1		
Splenic infarction	2	2		
Splenic artery bleeding	1	0		
Postoperative complications (%)	25 (24.3)	21 (25.3)	0.026	0.872
Anastomotic hemorrhage	2	3	0.492	0.483
Abdominal hemorrhage	1	3	1.526	0.217
Infection or dehiscence of the incision	0	0	/	/
Lung infection	22	11	2.069	0.15
Intestinal obstruction	2	5	2.115	0.146
Anastomotic leakage	1	0	0.81	0.368
Leakage of the duodenum stump	0	0	/	/
Abdominal infection	2	0	1.645	0.2
Chyle fistula	0	2	2.509	0.113
Gastroparesis	2	1	0.157	0.692
Number of perioperative deaths (within 30 days after surgery)	0	0	/	/
Grade of morbidity (%)			0.072	0.788
I–II	22 (88.0)	19 (90.4)		
III–IV	3 (12.0)	2 (9.6)		
Mortality within 30 days after surgery	0	0	/	/

*P < 0.05 was considered statistically significant

### Comparison of long-term survival

**Fig. 6:**
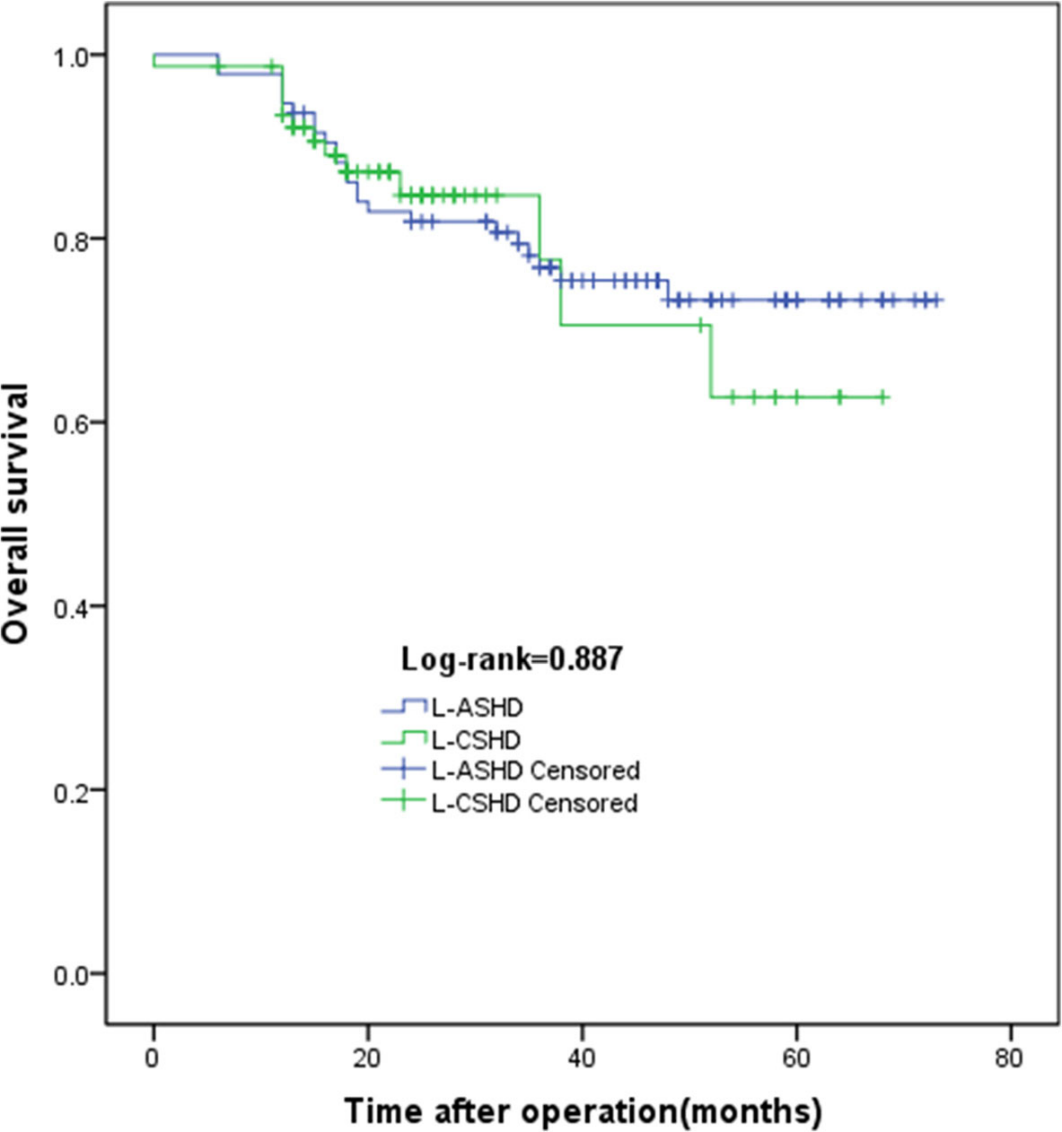
Kaplan–Meier analysis OS for L-ASHD and L-CSHD. There were no significant differences in overall survival between L-ASHD (blue line) and L-CSHD (green line) (χ^2^ = 0.02, *P* = 0.887)

**Fig. 7:**
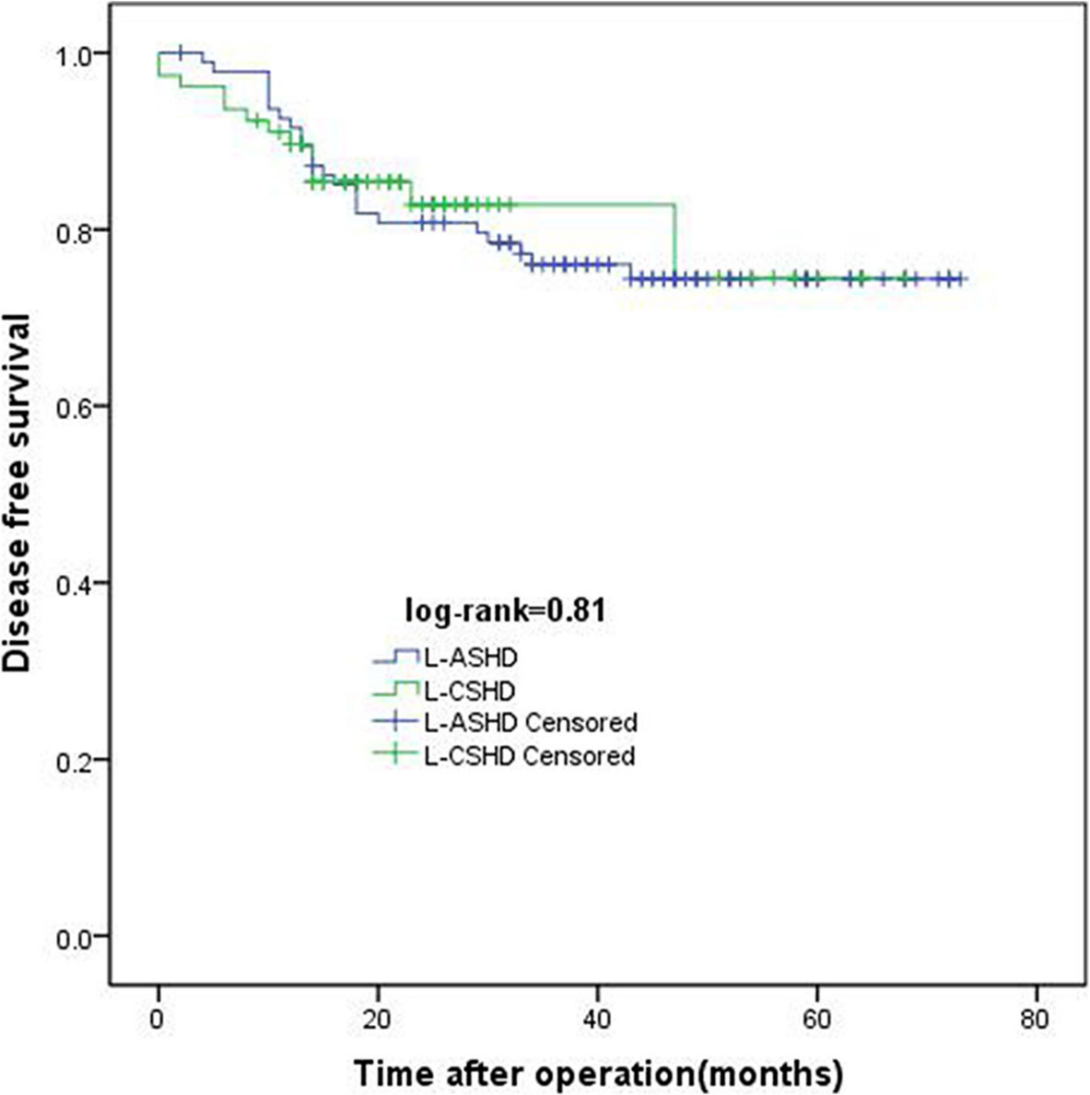
Kaplan–Meier analysis DFS for L-ASHD and L-CSHD. There were no significant differences in disease free survival between L-ASHD (blue line) and L-CSHD (green line) (*χ*^2^ = 0.058, *P* = 0.810)

We investigated long-term survival in the L-CSHD and the L-ASHD. All of the patients were followed up until the last visit or death. There was no difference noted between the two arms (*P* = 0.091). The 1-, 3-, and 5-year OS rates in the L-ASHD were 95%, 77%, 73%, respectively, which were not significantly different from those of the L-CSHD (93%, 77%, 63%, respectively). Kaplan–Meier survival curves showed that the L-CSHD had similar OS and DFS compared with those of the L-ASHD (Figs. 6 and 7).

We also investigated local recurrence and distant metastasis in the two groups. The results showed that there was no significant difference in the incidence of local recurrence and distant metastasis in the L-CSHD and the L-ASHD. Local recurrence after surgery mainly focuses on anastomotic recurrence or lymph node metastasis perigastric region whatever in the L-CSHD or in the L-ASHD. We noticed that there were two patients that suffered from splenic hilar lymph node recurrence and metastasis in the L-ASHD. However, we did not find it in the L-CSHD. The distant metastasis after surgery mainly focuses on the liver, lung, bone, and peritoneal implantation in the two groups (Table 4).

**Table 4:** Local recurrence and distant metastasis in the two groups

	L-ASHD *n* = 103	L-CSHD *n* = 83	*χ*2 value	*P* value
Local recurrence (%)	8 (7.77%)	8 (9.63%)	0.205	0.651
Anastomotic recurrence	5	2		
Splenic hilar lymph node recurrence and metastasis	2	0		
Lymph node metastasis in perigastric region of	4	7		
Non-splenic hilar region				
Distant metastasis (%)	14(13.59%)	11(13.25%)	0.005	0.946
Extensive pelvic and abdominal metastasis	1	0		
Peritoneal implantation	1	1	/	
Pelvic planting	0	1		/
Malignant ascites	1	0		
Liver	6	2		
Pancreas	0	1		
Spleen	0	1		
Lung	4	3		
Bone	2	2		
Brain	1	0		
Renicapsule	1	0		
Colon	1	2		
Uterus	0	1		
Kidney	1	1		
The lymph nodes outside the region	2	0		

*P < 0.05 was considered statistically significant

## Discussion

The traditional consensus for the treatment of advanced gastric cancer includes complete resection of the primary gastric cancer lesions and systemic lymph node dissection; such treatment can achieve the goals of radical treatment. However, numerous studies have confirmed that many patients with gastric cancer experience local recurrence after radical resection plus D2 lymph node dissection [4–6]. In recent clinical reports, examination of pathological specimens has demonstrated that cancer tissues are often present in the fatty and connective tissues present in the gastric cancer specimens and that the fatty and connective tissues containing the cancer tissues are surrounded by a common mesentery, the mesogastrium. Traditional D2 lymph node dissection is based on blood vessel anatomy, and ligation of the blood vessels is essential. Thus, during this procedure, the mesogastrium is incised, resulting in the spread of tumor tissues within the mesogastrium to the abdominal cavity. Based on these studies, Gong (in China) suggested that metastasis within the mesogastrium is an important factor causing local recurrence and proposed the “fifth metastasis route” hypothesis of gastric cancer [7, 8]. Taking into account domestic and international studies of mesenteric anatomy, we have attempted to use mesenteric anatomy-guided laparoscopic spleen-preserving circumferential splenic hilar lymph node dissection for treatment of gastric cancer. After study and practice, we found that fused spaces between mesenteries and between mesenteries and organs exist at the splenic hilum. These fused spaces are natural avascular surgical planes that can be used to facilitate separation. Under laparoscopic visualization, the separation in these spaces can effectively reduce intraoperative bleeding and secondary injury. Following mesenteric anatomy guidance and surgical protocols can reduce the difficulty of lymph node dissection behind the splenic hilum and make it possible to perform complete, thorough, “three-dimensional” dissection of lymph nodes in the splenic hilum.

Domestic and international researchers have reached a consensus that advanced gastric cancer requires lymph node dissection in the splenic hilum [3]. According to the 14th edition of the *Japanese Classification of Gastric Carcinoma* (2010), splenic hilar lymph nodes include the lymph nodes around the splenic artery distal to the pancreatic tail, the splenic hilum, the origins of the gastric short arteries, and the first branch of the left gastroepiploic artery in the greater curvature [3]. Due to the complex anatomy of the splenic hilum, the splenic lobar vessels, and the blood vessels in the spleen pole, splenic rupture and massive hemorrhage may accidentally occur. Therefore, lymph node dissection is difficult and risky. The procedure of splenic hilar lymph node dissection is not available at many domestic and international centers. Even when it is available, missing positive lymph nodes is common due to the lack of criteria for complete dissection. Many domestic and international studies have confirmed that the complete dissection of splenic hilar lymph nodes is closely related to the prognosis of patients [9, 10]. Therefore, it is necessary to completely and thoroughly remove the lymph nodes of the splenic hilum in advanced proximal gastric cancer.

To date, two types of surgical procedure for splenic hilar lymph node dissection, i.e., lymph node dissection with and without spleen preservation, are available domestically and internationally. Splenic hilar lymph node dissection without spleen preservation is associated with surgical trauma and a high incidence of postoperative complications and mortality and failed to improve postoperative 5-year survival (35.6% vs. 42.2%, *P* = 0.622) [11, 12]. It has gradually been abandoned by most centers at home and abroad. In traditional open splenic hilar lymph node dissection, due to the deep position and ease of rupturing of the spleen and the complex anatomy of the splenic hilum and the splenic blood vessels, surgeons need to completely separate the spleen and the pancreatic body and tail and retract them out of the abdominal cavity to perform complete dissection of No. 10 lymph nodes. The procedure is difficult, and postoperative complications such as splenic torsion and spleen displacement are common. Therefore, No. 10 lymph node dissection with spleen preservation via laparotomy is difficult to perform routinely. The magnified visual field provided by the laparoscope and the cavitation effect of the ultrasonic scalpel can help the surgeon clearly visualize the splenic artery and its branches and efficiently complete lymph node dissection through the correct planes and the anatomical fascial spaces. Korean and Japanese surgeons were the first to successfully perform spleen-preserving laparoscopic No. 10 lymph node dissection for proximal gastric cancer [13, 14]. In China, Huang et al. successfully confirmed the safety and feasibility of laparoscopic spleen-preserving No. 10 lymph node dissection [15, 16]. In recent years, Son et al. demonstrated that laparoscopic spleen-preserving splenic hilar lymph node dissection for advanced proximal gastric cancer has short-term and long-term efficacy similar to that of laparoscopic hilum lymph node dissection without spleen preservation [16, 17]. Huang et al showed that laparoscopic spleen-preserving splenic hilar lymph node dissection had a significantly longer 3-year DFS (61.6% vs. 53.7%, *P* = 0.034) compared with none splenic hilar lymph node dissection for advanced proximal gastric cancer [17]. However, because the procedure of laparoscopic spleen-preserving No. 10 lymph node dissection is difficult and requires advanced surgical skills, it is available at only a few centers in China. Moreover, only anterior splenic hilar lymph node dissection is available at these centers; therefore, all splenic hilar lymph nodes may not be completely removed. As a result, positive lymph nodes in the posterior splenic hilum may be missed. We believe that laparoscopic lymph node dissection in the posterior splenic hilum is not a routine procedure for two reasons. First, laparoscopic lymph node dissection in the posterior splenic hilum has rarely been reported domestically or internationally. Further studies are needed to confirm its necessity. Second, the lymph nodes in the posterior splenic hilum are adjacent to important organs. These lymph nodes are attached anteriorly to the spleen vessels and their branches, posteriorly to the anterior renal fascia, and laterally to the spleen and the pancreatic tail. Their anatomical relationships are complex, and the operating space is limited. Uncontrollable bleeding may occur accidentally, and the rate of splenectomy may increase. Therefore, dissection of the lymph nodes behind the splenic vessels is considered an extremely difficult procedure. However, we often find enlarged lymph nodes behind the spleen hilum during surgery. Based on the principle of complete radical lymph node dissection during radical tumor resection, complete and thorough dissection of the lymph nodes around the splenic hilum is of utmost importance in advanced gastric cancer. Our study showed that 28 of 186 patients had positive splenic lymph nodes (positive rate 15.05%), consistent with the lymph node metastasis rate of 9.8% to 20.9% reported in the literature [2]. Positive splenic lymph nodes were identified in 19 patients (a positive rate of 22.3%) in the L-CSHD and in nine patients (a positive rate of 8.7%) in the L-ASHD. The postoperative complication rate was not significantly different in the two groups. Although the time required for lymph node dissection was longer in the L-CSHD, the use of laparoscopic circumferential splenic hilar lymph node dissection can reduce the incidence of missing positive splenic hilar lymph nodes without increasing the incidence of surgical complications. Therefore, we believe that mesenteric anatomy-guided laparoscopic spleen-preserving circumferential splenic hilar lymph node dissection is safe and necessary for advanced gastric adenocarcinoma. Long-term survival in the two groups was also compared, and the result showed that there was no significant difference in the OS and DFS between the two groups.

The key to successful completion of the dissection is the appropriate use of the strategy of laparoscopic spleen-preserving No. 10 lymph node dissection. We provided complete guidelines for laparoscopic circumferential splenic hilar lymph node dissection and proposed the “four-step procedure for laparoscopic spleen-preserving circumferential splenic hilar lymph node dissection for treatment of gastric cancer.” Use of the standard surgical procedure and identification of the correct anatomical planes are essential when performing posterior splenic hilar lymph node dissection and reduce the difficulty of the procedure. The key to the success of the operation is adherence to the protocol of laparoscopic spleen-preserving circumferential splenic hilar lymph node dissection. The spleen’s vulnerable texture means that it is at risk of injury. The small and variant splenic hilar blood vessels are located deeply and overlap with the pancreatic tail; therefore, the main obstacles to laparoscopic spleen-preserving splenic hilar lymph node dissection include difficult exposure under the laparoscope, difficulty in applying effective retraction, and difficulty in controlling bleeding. The pancreas is located in the joint of the transverse mesocolon and the gastroduodenal mesentery. During the development of the primitive mesentery, complex fused fascial spaces are formed at the junctions of the mesentery with surrounding organs. The posterior space caudal to the pancreatic tail is filled with loose connective tissues and is posteriorly bordered by the anterior renal fascia (covering the left renal superior pole and the adrenal gland); it is an avascular surgical plane and can easily be expanded. In the posterior approach to the pancreas, sufficient separation of the pancreatic tail and the posterior space of the splenic hilum is the key to mobilizing the splenic pedicle. The mobilized splenic pedicle is easily retracted. This technique thus solves the problem of visualization. Once this is done, it is easy to dissect the blood vessels and their branches. In this condition, even if the blood vessel or the spleen were to be damaged, bleeding would be easy to control because the mobilized splenic pedicle can be easily controlled to avoid massive blood loss.

## Conclusions

Laparoscopic spleen- and pancreas-preserving circumferential splenic hilar lymph node dissection via the posterior approach to the pancreas is safe and feasible. It can help avoid the incomplete dissection of positive lymph nodes. Following the mesenteric anatomical guidelines and the appropriate surgical protocol can greatly reduce the difficulty of this procedure. This surgical technique may provide a new technical option for laparoscopic treatment of advanced gastric cancer. Although, our study showed that patients in the L-CSHD had similar OS and DFS compared with patients in the L-ASHD. However, the number of patients analyzed and follow-up time was limited. Therefore more patients and a longer follow-up time in the future will be collected.
